# Optogenetic Modulation of Urinary Bladder Contraction for Lower Urinary Tract Dysfunction

**DOI:** 10.1038/srep40872

**Published:** 2017-01-18

**Authors:** Jae Hong Park, Jin Ki Hong, Ja Yun Jang, Jieun An, Kyu-Sung Lee, Tong Mook Kang, Hyun Joon Shin, Jun-Kyo Francis Suh

**Affiliations:** 1 Center for Bionics, Korea Institute of Science and Technology (KIST), Seoul, 02792, Korea; 2 Korea University of Science and Technology, Daejeon, 34113, Korea; 3 Department of Electronics Engineering, Ewha Womans University, Seoul, 03760, Korea; 4 Department of Physiology, SBRI, Sungkyunkwan University School of Medicine, Suwon, 16419, Korea; 5 Department of Urology, Samsung Medical Center, Sungkyunkwan University School of Medicine, Seoul, 06351, Korea

## Abstract

As current clinical approaches for lower urinary tract (LUT) dysfunction such as pharmacological and electrical stimulation treatments lack target specificity, thus resulting in suboptimal outcomes with various side effects, a better treatment modality with spatial and temporal target-specificity is necessary. In this study, we delivered optogenetic membrane proteins, such as channelrhodopsin-2 (ChR2) and halorhodopsin (NpHR), to bladder smooth muscle cells (SMCs) of mice using either the Cre-loxp transgenic system or a viral transfection method. The results showed that depolarizing ChR2-SMCs with blue light induced bladder contraction, whereas hyperpolarizing NpHR-SMCs with yellow light suppressed PGE_2_-induced overactive contraction. We also confirmed that optogenetic contraction of bladder smooth muscles in this study is not neurogenic, but solely myogenic, and that optogenetic light stimulation can modulate the urination *in vivo*. This study thus demonstrated the utility of optogenetic modulation of smooth muscle as a means to actively control the urinary bladder contraction with spatial and temporal accuracy. These features would increase the efficacy of bladder control in LUT dysfunctions without the side effects of conventional clinical therapies.

Lower urinary tract (LUT) plays an important role in two physiological functions: storing and emptying urine. These functions are controlled by a complex neural circuit and synergized activity of smooth and striated muscles of the LUT^1^,^2^. Because of its complex structure, LUT is susceptible to deterioration by various diseases and injuries, such as bladder outlet obstruction^3^, diabetic mellitus^4^, and spinal cord injury^5^, often resulting in LUT dysfunctions. Conventional clinical approaches dealing with LUT dysfunctions, including pharmacological treatments (e.g. anticholinergics, β3-adrenergic receptor agonists, botulinum toxin) or sacral nerve stimulation, however, have often shown suboptimal efficacy with various side effects due to the lack of the target specificity^6^,^7^. Given that the counter-acting mechanisms of detrusor and urethral sphincter muscles between storing and emptying processes of urine, a treatment for one process would more likely compromise the function of the other process. Furthermore, the emptying process is much shorter than the storing process, thus making temporal targeting of drug treatment for emptying urine very difficult. In order to properly resolve these problems, therefore, this study presents alternative approach to modulate bladder contraction with temporal and spatial target-specificity.

Optogenetics has been developed and widely used to modulate biological behaviors of various cells^8^. The most notable advantage of optogenetics is a target-specificity; an expression of opsins can be targeted to a group of cells of interest using specific promotors of the cells. It was shown that the membrane potential of neurons of interest can be modulated via a combination of channelrhodopsin (ChR2) or halorhodopsin (NpHR) and respective light illuminations, thus enabling to switch on or off the action potentials of the neurons with precise temporal and spatial control^9^,^10^. In this study, we proposed that an optogenetic modulation of the membrane potential of bladder smooth muscle cells would control the contractility of the bladder without any intervention of bladder-associated neural circuits.

## Results

### Depolarization-Induced Bladder Contraction by ChR2 Activation

**Figure 1:**
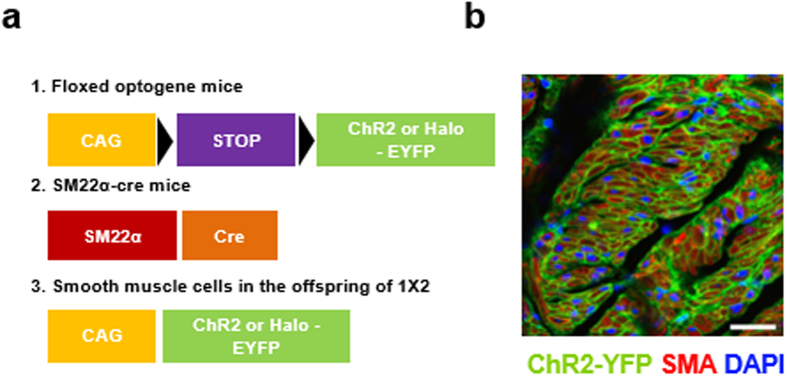
Transgenic expression of ChR2 in urinary bladder smooth muscles. (**a**) Schematics of expression of optogenes in bladder SMCs. Offspring (3) from breeding floxed optogene mice (1) with SM22α-cre mice (2) express optogene in their smooth muscle cells. (**b**) Confocal image of detrusor slice from the transgenic mice. YFP signals were detected in SMC (smooth muscle actin (red) and DAPI (blue)). Scale bar, 25 µm.

To verify the feasibility of the proposed approach, we obtained transgenic mice which express channelrhodopsin (ChR2) in the SMCs by crossbreeding the tagln-cre mouse line^11^ and the floxed ChR2 mouse line (ChR2_HR_)^12^ (Fig. 1a). ChR2 expression in the bladder SMCs of the resulting transgenic mice was confirmed by the intense EYFP signals colocalized with smooth muscle actin under a confocal microscope (Fig. 1b).

**Figure 2:**
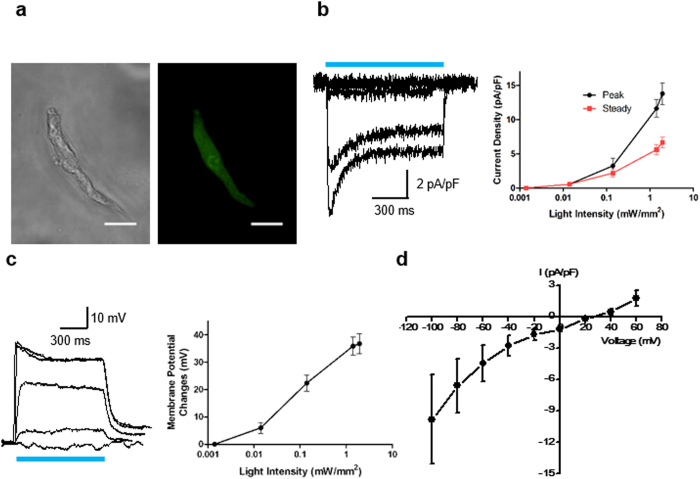
ChR2 activation induces inward current and membrane depolarization of smooth muscle cells. (**a**) Phase contrast and YFP imaging of ChR2-expressing SMC. Scale bar, 20 µm. (**b**) Representative traces of inward currents evoked by blue light at 0.0014, 0.014, 0.14, 1.41, 1.94 (mW/mm^2^). Relationship between inward currents versus light intensities (n = 5). (**c**) Representative traces of membrane potential changes evoked by blue light at 0.0014, 0.014, 0.14, 1.41, 1.94 (mW/mm^2^). Relationship between peak membrane potential changes versus light intensities (n = 5). (**d**) I-V curve of the photocurrents evoked by ChR2 (n = 5). The reversal potential was around + 20 mV. All error bars indicate SE.

Since the contraction of smooth muscles in the bladder is mediated by membrane depolarization^13^, we first assessed the depolarization of SMCs induced by ChR2 activation under blue light (473 nm). To this end, SMCs were enzymatically isolated from the ChR2-expressing transgenic mouse bladder (Fig. 2a), and tested on patch clamp (see supplementary methods). Blue light illumination immediately induced inward current in ChR2-expressing SMCs with an initial peak followed by quick relaxation to a plateau level (Fig. 2b, left), and the induced inward current density was strongly dependent upon the light intensity (Fig. 2b, right). At the onset of light, the resulting inward current depolarized membrane potential (Fig. 2c) with a similar dependency on light intensity. As the light intensity increased from 0.014 to 1.94 mW/mm^2^, the light-induced peak current density and peak membrane depolarization became saturated near 13.4 ± 2.4 pA/pF and 36.8 ± 3.7 mV, respectively. The current-voltage relationship (I-V curve) of the blue light-evoked current was obtained by a step-pulse protocol, and the current was reversed at around +20 mV (Fig. 2d). As the relative Na^+^ permeability of ChR2 is ~2-fold higher than K^+^ 14, the measured reversal potential (+20 mV) was close to the predicted value (+17 mV) by Goldman equation with P_Na_/P_K_ = 2, suggesting that the blue light-evoked current was purely mediated by ChR2.

### Light-Evoked Contraction of ChR2-Bladder: *Ex Vivo* and *In Vivo* Evaluations

**Figure 3:**
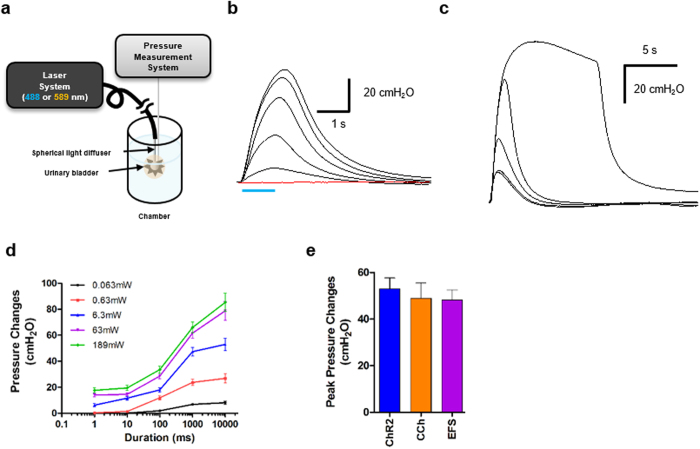
Blue light can mediate contraction of urinary bladder smooth muscles from transgenic ChR2-mice. (**a**) Schematics of experimental setup for optical stimulation and pressure recordings of a whole urinary bladder sample ex *vivo*: After animal was sacrificed, the bladder was isolated, instrumented with a polyethylene catheter along with a spherical light diffuser, and submerged in a organ bath of carbonated physiological saline. (**b**) Representative traces of pressure changes of whole bladder in response to 1 s of various light power stimuli (0.063, 0.63, 6.3, 63 and 189 mW). The red line is the control response of bladder from wild type mice (C57BL/6) to the maximum blue light (189 mW). (**c**) Representative traces of pressure changes of bladder in response to 63 mW light power with various illumination periods (1, 10, 100, 1000 and 10000 ms). (**d**) Relationship between the peak pressure changes of bladders and varying light parameters, the illumination period and the light power (n = 5). (**e**) ChR2-mediated bladder contractions (6.3 mW) were comparable to those induced by carbachol (3 μM) and EFS (50 V, 0.1 ms pulse duration and 20 Hz). All error bars indicate SE.

We next conducted intravesical pressure recording of a whole bladder *ex vivo* to characterize the contraction pressure change of the ChR2-bladder in response to light illumination. For *ex vivo* bladder test, the whole bladder was isolated from the transgenic ChR2-mouse of Fig. 1a, instrumented with a polyethylene catheter for pressure measurement along with a spherical light diffuser for optical stimulation, and submerged in an organ bath of carbonated physiological saline solution (Fig. 3a and see supplementary methods for details). Figure 3b shows that illumination with blue light evoked contraction pressure in the ChR2-bladder, whereas the age-matched wild type bladder did not respond to identical optical stimuli (red line in the figure), implying that the bladder contraction was primarily due to ChR2 activation. The bladder pressure change induced by optical stimulation was strongly dependent upon light intensity (Fig. 3b for the light power and Fig. 3c for the illumination periods), consistent with our patch clamp data (Fig. 2b and c) and the previous studies of ChR2-expressing neurons^10^: The peak contraction pressure change increased with greater light power (Fig. 3b and d) as well as longer illumination periods (Fig. 3c and d). The peak contraction pressure change with respect to the illumination intensity showed a sigmoid trend, in which the rate of pressure change decreased with the increase in light intensity (Fig. 3d). The peak contraction pressure change of the ChR2-expressing bladder by light (6.3 mW) was comparable to those by other stimulants such as carbachol (3 μM), a cholinergic agonist, and electrical field stimulation (50 VDC with 0.1 ms pulse duration at 20 Hz) (Fig. 3e), and was within the physiological range of voiding bladder pressure (40~50 cmH_2_O) of various species, including humans^1^ and rodents^15^.

**Figure 4:**
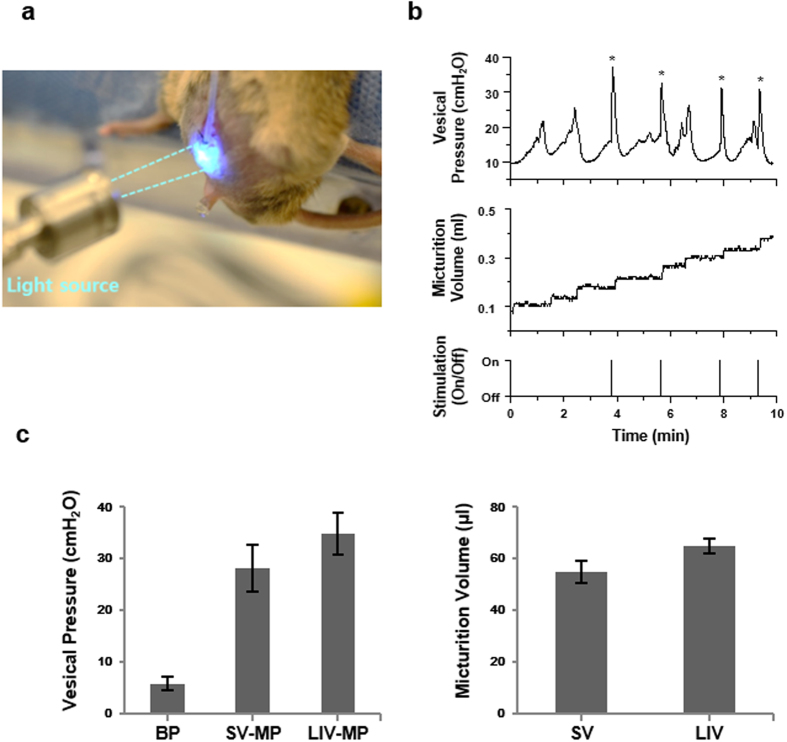
*In vivo* cystometry of light-activated urination of transgenic ChR2-mice. (**a**) Experimental setup: The anesthetized animal was subjected to *in vivo* cystometry evaluation and the voiding was induced by blue light illumination of 26 mW. (**b**) Representative trace of cystometry with transgenic ChR2-bladder. The vesical pressure elevation induced by optical stimulation was represented with asterisk. The pressure elevation without asterisk was a spontaneous voiding. (**c**) Cystometric parameters comparing spontaneous and light-induced voiding. (n = 6, BP; Baseline pressure, SV; Spontaneous voiding, LIV; Light-induced voiding, MP; Micturition pressure).

Furthermore, we conducted *in vivo* cystometry to test the ability of optogenetic bladder to discharge urine in response to blue light illumination. For *in vivo* cystometry, urethane-anesthetized mouse was fixed supine on a cystometry table after bladder catheterization with PE50 polyethylene tube. While being continuously infused with saline through the catheter, the exposed bladder was subjected to 1-s blue light stimulation of 26 mW at random instants, and the vesical pressure and micturition volume were monitored (Fig. 4a and see supplementary methods for details). Similar to the *ex vivo* results above, blue light stimulation of 26 mW caused the increase of intravesical pressure along with voiding of urine (Fig. 4b and Supplementary Movie 1), indicating that a light illumination can be used to discharge urine from ChR2-bladder *in vivo*. The result of cystometry (Fig. 4c) showed that while the baseline vesical pressure (BP) was 5.7 ± 1.3 cmH_2_O, the micturition pressures (MP) were 28.2 ± 4.5 cmH_2_O and 34.9 ± 4.0 cmH_2_O for spontaneous voiding (SV) and light-induced voiding (LIV), respectively, and that the micturition volumes were 54.9 ± 4.3 μl and 65.1 ± 2.8 μl for SV and LIV, respectively. While the average LIV-MP was higher than the average SV-MP with statistical significance (p < 0.01), the magnitudes of the MP and micturition volume data were within the normal range of cystometry data from urethane-anesthetized mice reported in literature^16^,^17^.

### Viral Transfection of ChR2 for Optogenetic Bladder Modulation

We next examined the efficacy of exogenous, instead of transgenic, opsins directly applied to wild type mouse bladders. To this end, we injected 10 μl of adenovirus containing Ad-CAG-hChR2 (H134R)-EYFP into C57BL/6J wild type bladder using a 32 gauge needle. At one week post-injection, the bladder function was examined both in *ex vivo* pressure recording (Fig. 3a) and *in vivo* cystometry (Fig. 4a).

**Figure 5:**
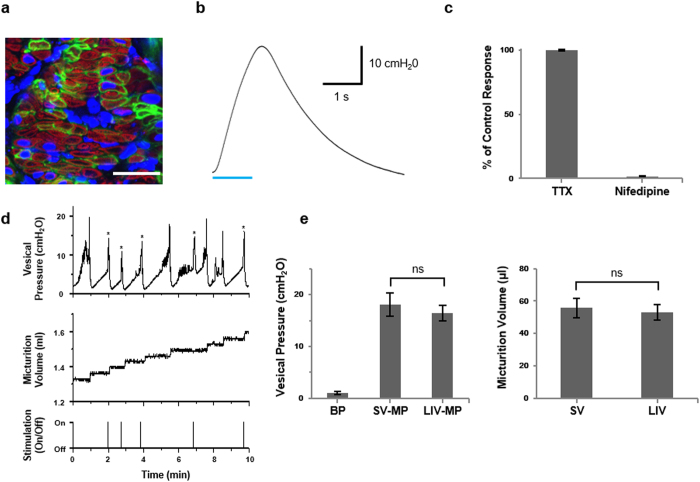
Urinary bladder with adeno virus-mediated ChR2(H134R) gene transfection. Bladders of C57BL/6J mice were injected with ad-CMV-hCHR2(H134R)-EYFP. (**a**) Cross-sectioned bladder (10 μm thick) stained with a-SMA antibody (red). Most of YFP was expressed in the membrane of SMCs (green) in the H134R-bladder. Nuclei were stained with DAPI in blue. Scale bar, 25 μm. (**b**) Representative trace of contraction pressure changes in H134R-bladder in response to 1 s continuous illumination of blue light *ex vivo*. At the bottom, blue bar indicates the blue light illumination for activation of ChR2(H134R) (473 nm, 63 mW and 1 s continuous illumination) (**c**) The effect of TTX (500 nM) and nifedipine (1 μM) on H134R mediated peak bladder contraction *ex vivo*. TTX did not affect on H134R induced bladder contraction (ns, repeated measures ANOVA (n = 5)). Nifedipine, on the contrary, totally abolished contraction of the same bladder (p < 0.001, repeated measures ANOVA (n = 5)). (**d**) Representative trace of *in vivo* cystometry evoked by blue light illumination of 26 mW. The vesical pressure elevation induced by optical stimulation was represented with asterisk. The pressure elevation without asterisk was a spontaneous voiding. (**e**) Cystometric parameters of virus-mediated mice comparing spontaneous and light-induced voiding. (n = 6, BP; Baseline pressure, SV; Spontaneous voiding, LIV; Light-induced voiding, MP; Micturition pressure) All error bars indicate SEM.

Expression of EYFP for ChR2 transfection was confirmed in the immunohistology of the mouse bladders at one week post injection (Fig. 5a). When subjected to blue light illumination in *ex vivo* experiment (473 nm, 63 mW for 1 s), the virally transfected ChR2-bladder samples demonstrated a sharp increase of contraction pressure (Fig. 5b), a similar pattern to what we saw with the transgenic ChR2-bladder samples (Fig. 3b). The average peak contraction pressure of virally transfected ChR2-bladder was 26.4 ± 2.2 cmH_2_O (n = 5), noticeably lower than that of the transgenic ChR2-bladder (Fig. 3e). This could be presumably due to incomplete ChR2-transfection over the entire smooth muscle volume of the bladder or to a possible inflammatory damage of bladder structure by needle injection of adenovirus. As a nonspecific CAG promotor was used in the viral construct, we also clarified a possibility of non-muscle origin of the light-induced contractile responses of the bladder. As shown in Fig. 5c, when pharmacologically treated with TTX (500 nM), a sodium-channel blocker, prior to blue light illumination, the contraction of the virus-transfected ChR2-bladder was not affected by TTX in response to blue light, indicating that ChR2-mediated bladder contraction was unrelated to nerve-mediated responses. When treated with nifedipine (1 μM), an L-type calcium channel blocker, however, the bladder contraction by blue light was completely abolished. It was thus confirmed that ChR2-evoked bladder contraction is mainly induced by the influx of calcium ions through voltage-gated L-type calcium channels of bladder SMCs with no neural mediation.

The result of *in vivo* cystometry (Fig. 5d and e) showed that average magnitudes of BP, SV-MP and LIV-MP were 1.1 ± 0.3 cmH_2_O, 18.1 ± 2.2 cmH_2_O, and 16.5 ± 1.5 cmH_2_O, respectively, which were noticeably lower than those of transgenic mice presented in Fig. 4c, consistent with the trend of *ex vivo* contraction pressure data described above. The micturition volumes were 55.7 ± 6.0 μl and 53.0 ± 4.7 μl for SV and LIV, respectively.

### Various Illumination Protocols for ChR2 Activation in Bladder Contraction

The voiding period in the normal micturition cycle is usually between 30~60 s^18^, during which contraction of the bladder smooth muscles needs to be maintained for complete voiding. In order to apply our ChR2-driven optogenetics to the urinary bladder, it may be necessary for the optically driven contraction of the ChR2-bladder SMCs to be maintained for the entire voiding phase. A continuous optical stimulation of the ChR2-bladder for such a prolonged period, however, may not be practical in clinical applications of the optogenetics for urinary bladder dysfunctions, because prolonged optical stimulation with a short wavelength light may cause potential detrimental effects such as phototoxicity^19^ and thermal damage to the cells^20^. In this study, therefore, we tested the feasibility of repeated pulsed illuminations of ChR2-bladder as a means to shorten the illumination period necessary to maintain the bladder contraction using *ex vivo* contraction recording protocol presented in Fig. 3a.

**Figure 6:**
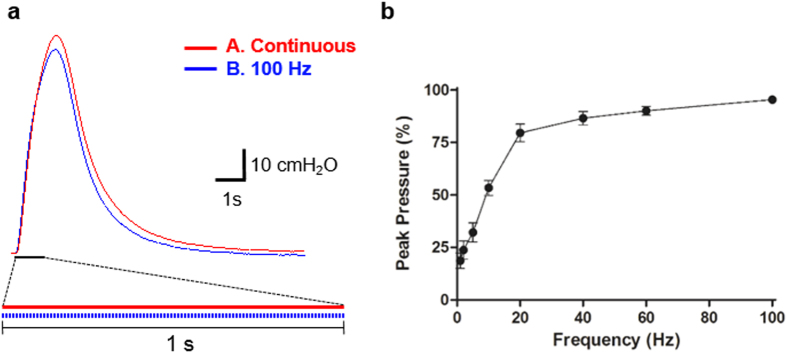
Transgenic ChR2(H134R)-bladder subjected to continuous and pulsed optical stimulation protocols for 1 s *ex vivo*. (**a**) The pressure traces to a short-term illumination (at 63 mW) for 1 s using two types of illumination protocols: a continuous illumination for 1 s (red line) and a pulsed illumination at 100 Hz (the pulse width = 1 ms, duty ratio = 10%) (blue line). Note that the power requirements for the pulsed illumination at 100 Hz is one tenth of that for the continuous illumination. (**b**) The peak pressure magnitude in response to the pulsed illumination at varying frequency is represented as the percent ratio with respect to that by the continuous illumination for 1 s, showing a sigmoid trend as the pulse frequency increases (n = 5). All error bars indicate SE.

We first subjected ChR2-bladder samples isolated from the aforementioned transgenic mice (Fig. 1) to optical stimulation for 1 s using either a continuous or repeated, pulsed (100 Hz, pulse width = 1 ms) illumination of 63 mW blue light (red line and blue dotted line, respectively, at the bottom of Fig. 6a). Both protocols produced a similar level of contraction pressure in the bladder (Fig. 6a), and the peak contraction pressure magnitude in response to the pulsed illumination had a sigmoid trend as pulse frequency increased, with the peak contraction pressure from 100 Hz pulsed illumination being 92.4 ± 0.2% of that by continuous one (n = 5, Fig. 6b). This result suggests that a 100 Hz pulsed protocol could produce a bladder contractile profile similar to that of a continuous illumination, but with only one tenth of the total illumination period.

**Figure 7:**
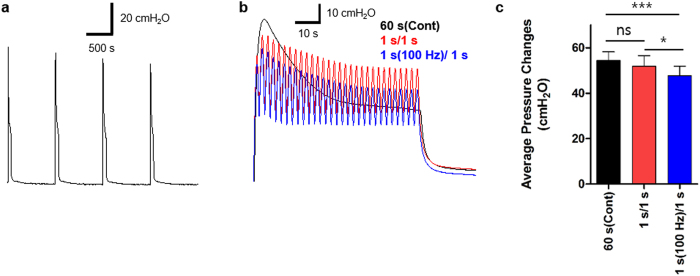
Transgenic ChR2(H134R)-bladder subjected to continuous and pulsed optical stimulation protocols for 60 s *ex vivo*. (**a**) Representative traces of bladder pressure evoked by repeated light illuminations of 60 s-ON with 840 s-OFF. Blue bar indicates the stimulation with blue laser (473 nm). (**b**) An isolated bladder contraction over the illumination period of 60 s (red box in (a)) was compared among three different optical protocols using blue laser with 63 mW power: a continuous illumination for 60 s (energy emission = 3.78 J, black), an intermittent illumination with ON for 1 s and OFF for 1 s (energy emission = 1.89 J, red), and an intermittent, pulsed illumination with 100 Hz for 1 s and OFF for 1 s (energy emission = 0.19 J, blue). All three protocols produce a relaxation of the peak pressure over the illumination period of 60 s. (**c**) The average pressure changes over the 60 s illumination period between three illumination protocols. Pressure changes to the 100 Hz protocol are slightly lower than those to the continuous one (p < 0.001, repeated measures ANOVA (n = 5)). All error bars indicate SE.

We next simulated the 60 s-voiding phase of the micturition cycle using a ChR2-bladder. When the bladder sample was exposed to a series of 60 s continuous illuminations with 840 s intervals, the peak contraction pressure induced by each individual optical stimulus was nearly maintained over the repeated stimulation (Fig. 7a). For each stimulus (dotted red box in Fig. 7a), the onset of light caused bladder pressure to quickly rise to an initial peak followed by a steady relaxation to a plateau level during illumination, and the bladder pressure quickly decayed to the baseline level at the cessation of illumination. We then applied the aforementioned 1 s-pulsed protocols to the 60 s-voiding phase, and compared it with a continuous illumination for 60 s (Fig. 7b). Continuous illumination with a blue laser of 63 mW for 60 s (black) emitted 3.78J of energy in total. The total energy emitted by an intermittent optical stimulation with ON for 1 s and OFF for 1 s for a total of 60 s was 1.89 J (red), whereas that by an intermittent, pulsed illumination with 100 Hz for 1 s and OFF for 1 s was 0.19 J (blue). The overall average pressure changes evoked by the three optical stimulation protocols were 54.5 ± 3.8, 51.9 ± 4.7, and 47.5 ± 4.3 cmH_2_O, respectively, where the pressure change in response to 100 Hz illumination was slightly lower than the other two illumination protocols (p < 0.001, n = 5) (Fig. 7c). So, when applied to practical modulation of bladder, intermittent stimulation which requires less energy seems more appropriate for the energy requirement issues in the future.

### Comparison between ChR2(H134R) and ChR2(C128S/D156A) for Bladder Contraction

Another way to reduce input energy for optical stimulation is a choice of the optimal opsin in modulating bladder contraction. While we found that pulsed blue light illumination could be more efficient than continuous illumination in terms of energy emission for H134R-ChR2 (Figs 6 and 7), the use of C128S/D156A, a ChR2 variant that is a stable step-function opsin (SSFO) with a slow off-kinetic decay time (~30 min)^21^, might be an effective choice to mediate bladder contraction *in vivo*. It has been reported that pulsed blue light illumination causes SSFO-ChR2 channels to remain activated until a yellow light is applied to deactivate the channels^21^. We expect that this feature would allow us to use brief pulsed illumination to switch ChR2 channels on and off in the bladder and to reduce energy requirement for opsins.

**Figure 8:**
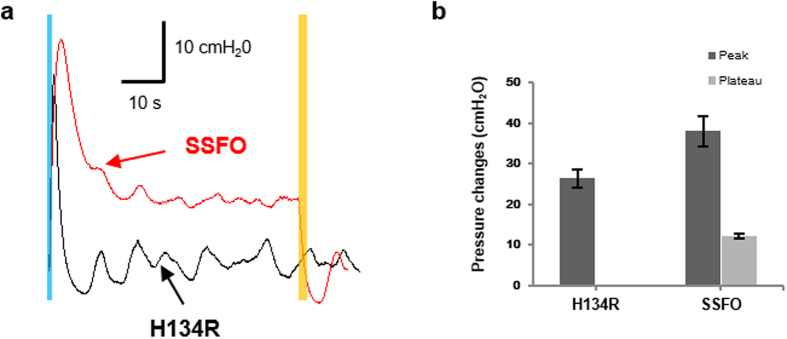
Comparison of bladder function between ChR2(H134R) and SSFO(C128S/D156A) gene transfections. Bladders of C57BL/6J mice were injected either with ad-CMV-hCHR2(H134R)-EYFP or ad-CMV-hCHR2(C128S/D156A). (**a**) Representative traces of pressure changes in H134R-bladder (black) and SSFO-bladder (red) in response to 1 s continuous illumination of blue light. At the onset of blue light illumination, H134R-bladder contraction quickly rose to a peak pressure, then decayed to the baseline level (black), whereas SSFO-bladder produced an initial peak pressure followed by a reduced, but elevated plateau pressure until yellow illumination deactivated the SSFO (red). Blue bar indicates the blue light illumination for activation (473 nm, 63 mW and 1 s continuous illumination) and yellow bar, the yellow light illumination for deactivation of SSFO (589 nm, 189 mW and 2 s continuous illumination). (**b**) The average peak and plateau pressure changes after 1 s illumination (n = 5). All error bars indicate SE.

When examined with *ex vivo* contraction pressure recording setup (Fig. 3a), the SSFO-bladder samples, subjected to blue light (473 nm, 63 mW for 1 s), demonstrated an initial peak contraction pressure (38.0 ± 3.6 cmH_2_O, n = 5) followed by an elevated level of plateaued pressure (12.2 ± 0.6 cmH_2_O, n = 5) for 60 s until illumination with a yellow light (589 nm, 189 mW for 2 s) (red line), as shown in Fig. 8. It was significantly distinctive against the contraction pressure of H134R-bladder samples which showed initial peak contraction pressure (26.4 ± 2.2 cmH_2_O, n = 5) followed by quick decay to a baseline level (black line). Please note that the sustained plateau-pressure magnitude of SSFO-bladder was significantly lower than the bladder contraction pressure magnitudes of all our H134R-bladder experiments *ex vivo* as well as *in vivo*. This result confirmed that, with the aid of a viral vector, SSFO opsin can be exogenously delivered directly into the bladder smooth muscles and can control the bladder contraction in an energy-efficient manner.

### Ameliorating Overactive Bladder via Halorhodopsin Activation

**Figure 9:**
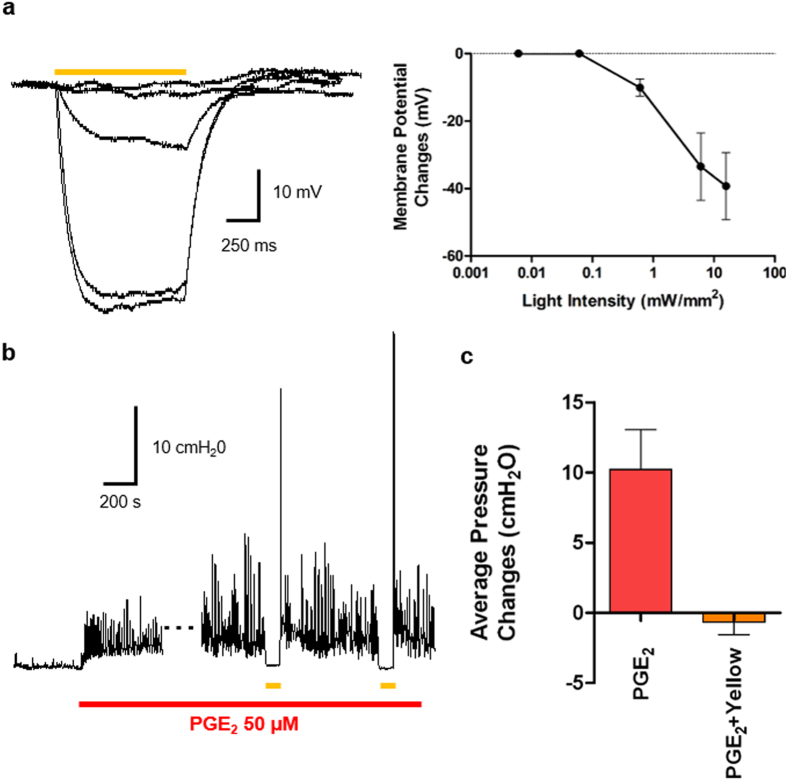
Hyperpolarization of SMC by yellow light activation can ameliorate PGE_2_-induced overactivity of NpHR-bladder. NpHR-bladder was produced transgenically (Fig. 1a) and subjected to patch clamp test (Fig. 2a) and *ex vivo* evaluation (Fig. 3a). (**a**) Representative traces of membrane potential changes of NpHR-SMCs evoked by yellow light (589 nm) stimulation at 0.006, 0.06, 0.6, 6, and 16 mW/mm^2^ (left). Yellow bar indicates yellow illumination for 1 s. Membrane potential changes evoked by yellow light were strongly dependent upon yellow light intensity (right, n = 5). (**b**) PGE_2_ (50 μM) treatment induced an overactive bladder indicated by increase of spontaneous bladder contraction. The spontaneous bladder contraction was suppressed by yellow light (189 mW) illumination for 60 s. (**c**) Average pressure changes of the NpHR-bladder were compared between the bladders with PGE_2_ treatment and with PGE_2_ treatment and yellow light illumination (n = 5). Average pressure change was measured with respect to the average intravesical pressure of bladder sample prior to the PGE_2_ treatment. Yellow light significantly reduced the increased bladder pressure evoked by PGE_2_ treatment (p < 0.01, repeated measures ANOVA n = 5). All error bars indicate SE.

We next tested the feasibility of halorhodopsin (eNpHR3.0)^22^ as a tool to suppress overactive bladder symptom (OAB), a frequent phasic contraction of the urinary bladder regardless of voiding. To this end, we first produced a transgenic mouse model with an NpHR-expressing bladder using a procedure similar to the ChR2-bladder model (Fig. 1a). Using a voltage-clamp method, we confirmed that yellow light (589 nm) illumination hyperpolarized the membrane of the isolated NpHR-SMCs (Fig. 9a). The yellow light-induced potential change showed a sigmoid trend as the light intensity increased.

We then applied prostaglandin E_2_ (PGE_2_, 50 μM) to an NpHR-bladder sample isolated from the above transgenic mice to simulate OAB^23^ in *ex vivo* contraction pressure recording protocol (Fig. 3a), which was confirmed by an increase in spontaneous bladder contraction after drug treatment (Fig. 9b). The PGE_2_ treatment increased the average intravesical pressure of bladders to 10.22 ± 2.8 cmH_2_O, while yellow light (189 mW) suppressed the evoked intravesical pressure to −0.67 ± 0.8 cmH_2_O, and the optogenetic suppression was repeatable. This result showed that hyperpolarization by chloride pump could cancel out the depolarization mediated by PGE_2_ treatment, indicating that hyperpolarization of the SMC membrane with halorhodopsin could be used to ameliorate OAB symptoms. At the end of yellow light stimuli, a transient rebound contraction was observed (Fig. 9b).

## Discussion

In this study, we first introduced the idea that optogenetic modulation of membrane potential of SMCs could successfully regulate the contractile behaviors of the urinary bladder, bi-directionally evoking and inhibiting bladder contraction depending on the types of opsins. Depolarization of SMCs by ChR2 directly contracted the detrusor, while hyperpolarization of SMCs with NpHR suppressed overactive bladder contraction. These findings suggest that optogenetics can be applied for the treatment of LUT dysfunctions such as OAB and DUA. It has been recently reported that contraction of blood vessels can be also regulated by ChR2^24^.

Our approach to regulating urinary bladder contraction via optogenetic modulation on SMCs has several advantages over other current strategies such as pharmacological treatment and sacral nerve stimulation. First, because the neural circuits involved in the storage and voiding of the urinary bladder interact with other neural functions in a complex manner, almost all of the current gold-standard nerve-targeted treatment approaches fail to specifically address isolated neuro-circuits, causing undesirable side effects such as unwanted bowel movements or sexual function^1^. Electrical stimulation of the efferent nerves to the bladder (sacral anterior roots) can produce a simultaneous contraction of both the detrusor and urethral sphincter muscle as well as the skeletal muscles in the lower extremities^25^. As the smooth muscles are the end target of urinary bladder modulation, we believe that applying optogenetics directly to bladder SMCs allows us to exclusively manipulate the contractile behavior of the bladder without compromising other muscle functions.

Second, unlike pharmacological strategies, the present optogenetic approach allows for temporal control of the contractile behaviors of the bladder using an on-off switch for illumination. The ability to temporally modulate bladder contraction would be especially crucial for the treatment of patients with DUA, as the voiding phase is a relatively short process that takes less than one minute. Such a temporally targeted therapy is very difficult to achieve with oral administration of pharmacological agents.

We showed the feasibility of optogenetic control of bladder in both transgenic and virally transfected animal models, suggesting a potential for future clinical trials in human patients with LUT dysfunctions. However, two major issues need to be addressed towards its clinical application. First, the stable, long lasting and safe expression of opsins in detrusor needs to be guaranteed. While the present study showed optogene transfection to the bladder using adenovirus, this virus is significantly limited in human clinical trials due to its transient gene expression and high immunogenicity^26^,^27^. Instead, adeno-associated viruses with more stable gene expression and low immunogenicity^28^ have been widely used in human clinical trials^29^,^30^,^31^, and may represent a better choice for optogenetic bladder modulation in future human trials^32^,^33^.

Second, the size of bladder could be also an important issue for future human application of our approach. As the surface area of human bladder is approximately hundreds times larger than that of mouse bladder, the total amount of optical energy required to activate the entire opsins-SMCs in human bladder could become too large for a practical application. Our optogenetic approach may require an implant consisting of light source and power battery, where effective power management would become a potentially critical issue to conserve battery power. In this study, we showed that an intermittent, pulsed illumination could produce a bladder contraction sufficient enough for effective voiding, yet emit considerably less energy compared to continuous illumination. We also showed that SSFO opsin with slow off-kinetics^21^ could allow us to use brief pulses of light to switch bladder contraction on and off, further reducing energy emission. These approaches will help not only reducing potential phototoxicity^19^ and thermal^20^ damage to the cells but conserving the battery power.

The present study demonstrated that NpHR could inhibit the contraction of bladder SMCs. While NpHR can be used to suppress OAB symptoms, it could also lead to accumulation of excessive Cl^−^ in the SMC cytosol, a nonphysiological condition. It was previously reported that a rebound potential was observed when neurons expressing NpHR were illuminated with yellow light^34^. While a rebound potential was not observed after isolated NpHR-SMCs were subjected to yellow light illumination (Fig. 9a) in our study, the whole bladder samples during intravesical pressure recordings showed a slight rebound contraction after yellow light stimulation (Fig. 9b) and its amplitude increased as the illumination time became longer (data not shown). Moreover, we observed that the PGE_2_ treated bladders were more sensitive to the termination of yellow light, and this rebound contraction was reduced by nifedipine treatment (data not shown). Recently, a blue-light–induced K + channel 1 (BLINK1) was introduced^35^ that exploits the potassium equilibrium to hyperpolarize the membrane potential that acts like BK channels in bladder SMCs^36^. This channel might be an alternative for alleviating the overactive symptom of SMCs without any nonphysiological disturbance of the cells such as excessive accumulation of Cl^−^ in the cytosol.

Although the present study focused mainly on modulation of detrusor contraction, a complete modulation of urination can be achieved only when both contraction and relaxation of the detrusor and urethral sphincter muscles are properly synchronized. Recently the feasibility of optogenetic on skeletal muscle has been introduced^37^, suggesting that the sphincter can be also modulated by optogenetics, which will allow a synchronized modulation of the detrusor and sphincter using both ChR2 and NpHR.

## Methods

All animal experiments and procedures were approved by the Institutional Animal Care and Use Committees of the Korea Institute of Science and Technology and Samsung Medical Center, and all experiments were conducted in accordance with the relevant guidelines and regulations set by the Committees.

### Transgenic Animal Models

Specific expression of opsins in SMCs of mice models were achieved via a Cre-loxp system. For the transgenic expression of ChR2 (H134R) in bladder smooth muscles of mice, we crossbred Tagln-cre mice (B6.Cg-Tg (Tagln-cre) 1Her/J, Jackson Lab stock #6878) with mice containing floxed ChR2 (H134R)/EYFP fusion protein (B6;129S-Gt (ROSA) 26Sor < tm32.1 (CAG-COP4*H134R/EYFP) Hze, Jackson Lab stock #12569). For NpHR expression, mice containing floxed Halo (eNpHR 3.0) (B6;129S-Gt (ROSA) 26Sor tm39 (CAG-hop/EYFP) Hze, Jackson Lab stock #14539) were used instead. The offspring from this mating showed abundant expression of ChR2 and eNpHR proteins, respectively, in the membranes of the bladder SMCs. The mice were provided with water and food ad libitum and reared under a 12-hour light/dark cycle. The mice were sacrificed by intraperitoneal injection of urethane (1.5 g/kg) followed by cervical dislocation. Ureters were ligated with a silk thread and the bladder with distal urethra was harvested for subsequent experiments.

### Adenoviral Constructs and Animal Transfection

In order to achieve expression of exogenous hChR2 (H134R) and hChR2 (C128S/D156A) opsins in the mouse bladder, we injected adenoviral vectors packaged with respective opsins directly into the detrusor muscles of wild type mouse bladders.

*In vivo* grade Ad-CMV-hChR2 (H134R)-EYFP and Ad-CMV-hChR2 (C128S/D156A) viral constructs with a type 5 (dE1/E3) backbone were titrated at 1.8 × 10^12^ VP/ml and 2.6 × 10^12^ VP/ml, respectively (Vector Biolabs). Nine-week-old male mice (C57BL/6J) were anesthetized by inhalation of 2% of isoflurane (vol/vol), and body temperature was maintained at 37 °C using a rectal-probe-coupled heating pad (JD-OT-06DT, JEUNGDO B&P, Korea). After the abdominal cavity area was shaved and disinfected with povidone iodine, a minimum laparotomy was performed along the abdominal line to expose the bladder. A total volume of 11 μl, consisting of 10 μl of the virus solution and 1 μl of fast green dye dissolved in PBS for visualization of injection, was carefully injected into the region between the serosa and detrusor layer using a 10 μl Hamilton syringe and 33-gauge needle (#7803-05, Hamilton). The injection was divided into 4~5 injections of 2~3 μl each to completely cover the entire bladder surface. After virus injection, the incision was closed with a 6–0 silk ligature and disinfected with povidone iodine. Antibiotics (Cefazolin sodium (0.44 ml/kg)) and anti-inflammatory drugs (Methampyrone (0.2 ml/kg)) were administered into the thigh muscle after surgery. The mice were provided with water and food ad libitum and reared under a 12-hour light/dark cycle. At one-week post-surgery, each animal was sacrificed and the bladder with urethra was harvested for subsequent experiments.

### Single SMC Dissociation and Patch Clamp

All electrophysiology data were acquired from conventional whole cell patch clamp recordings of dissociated single SMCs at room temperature. To dissociate single SMCs from the animal bladder, a bladder sample immediately after harvest was perfused in Ca^2+^ free solution (80 (mM) monosodium glutamate, 55 NaCl, 6 KCl, 2 MgCl_2_, and 10 Glucose, 10 HEPES, pH 7.3~7.4 adjusted with NaOH) at room temperature. Urothelium, lipid and connective tissue were carefully removed from the bladder surface using fine scissors. The remaining bladder sample was cut into several pieces and incubated in 2 ml of Ca^2+^ free solution with 1 mg/ml papain (#4762, Sigma), 1 mg/ml bovine serum albumin (#82–100–6, Millipore), and 1 mg/ml ditheioerythritol (#8161, Sigma) for 14 min at 37 °C. After incubation, the tissue samples were transferred to 2 ml of Ca^2+^ free solution with 1 mg/ml collagenase type 2 (#6885, Sigma), 1 mg/ml bovine serum albumin (Millipore), and 100 μM CaCl_2_ for 4 min at 37 °C. The enzymatically treated tissue samples were washed several times with Ca^2+^ free solution and gently triturated with a fire-polished pasteur pipette to dissociate single SMCs from the samples. A few drops containing single SMCs were placed on the recording chamber mounted on an inverted microscope (IX-70, Olympus) and perfused with the recording bath solution (mM) (145 NaCl, 5 KCl, 2 CaCl_2_, 1 MgCl_2_, 10 Glucose, 10 HEPES, pH 7.4 adjusted with NaOH) at 5 ml/min during patch clamp experiments.

Recording pipettes for patch clamp experiments were pulled from borosilicate micro pipette glass (#1B150F-4, World Precision Instruments) to give 2–3 MΩ tip resistance when filled with an internal pipette solution (mM) consisting of 140 KCl, 10 HEPES, 1 MgATP, and 5 EGTA, with pH adjusted to 7.2 with KOH. Pipette capacitance and series resistance were compensated up to >70%, and cell capacitance was measured after achieving the whole-cell configuration. For current clamp recordings, the initial cell membrane potential was set at −50 mV by manipulating the current injection. Patch clamp signals were acquired, amplified using a patch clamp amplifier (Axopatch 1D, Axon Instruments) and controlled by pClamp software 7.0 (Axon Instruments). The signals were filtered at 5 kHz and recorded at 10 kHz. Recorded traces were analyzed with Origin 8 software (Microcal Inc).

### Contraction Pressure Recording of Whole Bladder Samples *Ex Vivo*

After lipid and connective tissues were removed from the isolated bladder, the bladder cavity was washed twice with PBS solution. Both a polyethylene catheter (ID 0.58mm, OD 0.965mm, Becton Dickinson) and spherical diffuser (600 μm core-diameter, 0.37NA, SD200, Medlight, Switzerland) were inserted into the bladder via the remaining urethra and tied with a silk thread to prevent any leakage through the urethra. The instrumented bladder sample was submerged in a 10 ml organ bath containing carbonated physiological saline (mM) (119 NaCl, 4.7 KCl, 24 NaHCO_3_, 1.2 KH_2_PO_4_, 2.5 CaCl_2_, 1.2 MgSO_4_, and 11 Glucose, pH = 7.3~7.4) at 37 °C. The catheter was connected via a 3-way valve to a syringe pump (PHD 2000, Harvard Apparatus Ltd.) and to a pressure transducer (BLPR2, World Precision Instruments) for intravesical pressure measurement. The bladder was carefully filled with 0.9% saline (room temperature) at the rate of 0.07–0.09 ml/min, up to final volume of 0.1–0.15 ml, and equilibrated for at least 1 hour before the experiment. In order to check the contractile viability of the whole bladder, the bladder was briefly exposed to 3 μM carbachol, and washed twice with physiological saline after the maximum pressure was achieved. The pressure data was amplified with a transducer amplifier (TBM4M, World Precision Instruments) and acquired and processed at 200 Hz sampling rate using data acquisition system (Digidata 1550a, Molecular Devices).

### Optical and Electrical Field Stimulations of Whole Bladder Samples *Ex Vivo*

The light sources were diode-pumped solid-state lasers (CNI Optoelectronics Tech, China) with wavelengths of 473 nm (blue, MBL-F/300 mW) and 589 nm (yellow, MGL-N/500 mW). These lasers were combined through a polarizing beam splitter (PBS) for the multi-wavelength stimulation. Laser power was modulated by a variable ND filter (NDC-50C-4M-B, Thorlabs), and temporal parameters such as frequency and duration were regulated by a function generator and shutter system (SR470, Stanford Research Systems). The end of the optical fiber was connected to a spherical diffuser (SD200, Medlight, Switzerland) in order for the laser beams to uniformly scatter over the entire spherical surface inside the bladder sample.

Electrical field stimulation (EFS) was induced with a custom-built DC electrical stimulator and delivered to the bladder samples using platinum electrodes. The stimulation parameters of EFS were 50VDC, 0.1ms pulse duration, and 20 Hz, as previously described^15^.

Bladder samples were also treated with exogenous drugs: tetrodotoxin citrate (TTX) (#1069, Tocris, Bristol, UK) carbarchol (CCh) (#C4382, Sigma) and nifedipine (#N7634, Sigma). TTX was used to block nerve-mediated bladder contraction, CCh to stimulate M3 muscarinic receptors which play a major role in contraction of bladder smooth muscles, and nifedipine to block voltage-gated L-type calcium channels. We used PGE2 to induce overactive symptom of bladders. PGE2 was added to the bath to make final concentration (50 μM).

### *In Vivo* Cystometry and Voided Urine Measurement

The animals were anesthetized by urethane (1.2 g/kg, S.C.), and maintained at room temperature. After bladder was exposed by the same way described above (virus injection section), a small incision was made around bladder dome to insert polyethylene catheter (PE50). The anesthetized animal was then fixed supine on a table, which was positioned vertically so that the exposed bladder faced front toward the light source and that the urethra meatus of the animal pointed downward. A plastic cup was filled with filter paper for accurate measurement of voided urine^16^, placed underneath the urethra meatus, and connected to a force transducer for weight measurement (FT0314618, Natus). During the cystometry, the bladder was continuously infused with saline at the rate of 1.5 ml/h through the PE50 catheter with the bladder pressure being monitored (P23XL, Ohmeda Medical Devices Division). For optical stimulation, the exposed bladder was subjected to 1-s blue light (473 nm) stimulation of 26 mW (i.e., approximately 0.9 mW/mm^2^) at random instants. The output signals of both cystometry and weight of voided urine were acquired and processed at 200 Hz sampling rate using data acquisition system (PowerLab 8/30, AD Instruments).

### Immunohistochemistry

Isolated whole bladders were pre-fixed in 4% paraformaldehyde overnight and cryoprotected in 30% sucrose. The samples were embedded in OCT compound (Leica Microsystems, Germany), cryosectioned into 10
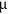
m thick slices, and mounted on microscope slides. After permeabilization in a solution containing 0.5% triton X-100 and 6% bovine serum albumin for 1 hour, the sections were incubated overnight at 4 °C with primary antibody [Anti-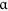-smooth muscle actin (#ab21027, Abcam), 1:200]. The sections were then washed three times with 1X PBS and incubated with the secondary antibody [Alexa Fluor 594 Donkey Anti-Rabbit IgG (#A21207, Invitrogen), 1:1000] for 1 hour at room temperature. The sections were subsequently washed three times with PBS and stained with DAPI (#R37606, Molecular Probes) to visualize the nuclei. Finally, the sections were rinsed with PBS three times and mounted with Clearmount (#008010, Invitrogen, UK). Confocal fluorescence images were acquired using a ZEISS scanning laser microscope with a 20X or 40X oil immersion objective lens (CLSM780, ZEISS, Germany).

### Data Analysis and Statistical Analysis

Data were analyzed by Origin8. The normality of data distribution was examined using the Shapiro-Wilk test with p > 0.05. All data groups were found to pass the null-hypothesis of normal distribution. All statistical data are indicated as mean ± s.e. (GraphPad Prism). Repeated ANOVA tests were used to compare the responses of bladders to stimulations (ChR2, CCh, and EFS), and pharmacological interventions (TTX and nifedipine). The statistical significance of the comparison was assessed by Tukey’s post-hoc analysis. As the sample size in each experimental group (n = 5) was relatively small, the statistical results were also double-checked using non-parametric Friedman ANOVA, and the results were the same as those by repeated ANOVA tests.

## Additional Information

**How to cite this article**: Park, J. H. *et al*. Optogenetic Modulation of Urinary Bladder Contraction for Lower Urinary Tract Dysfunction. *Sci. Rep.* **7**, 40872; doi: 10.1038/srep40872 (2017).

**Publisher's note:** Springer Nature remains neutral with regard to jurisdictional claims in published maps and institutional affiliations.

## Supplementary Material

Supplementary Video

Supplementary Information
