# Supplementary material for: Optogenetic Modulation of Urinary Bladder Contraction for Lower Urinary Tract Dysfunction
Source: Sci Rep. 2017 Jan 18;7:40872. doi: 10.1038/srep40872 (PMC5241665; doi:10.1038/srep40872)
Supplement: Supplementary Information [file srep40872-s2.pdf]

Optogenetic Modulation of Urinary Bladder Contraction for Lower Urinary Tract Dysfunction

Jae Hong Park[1†], Jin Ki Hong[1,2†], Ja Yun Jang[1,3], Jieun An[4],

Kyu-Sung Lee[5], Tong Mook Kang[4], Hyun Joon Shin[1,2],

and Jun-Kyo Francis Suh[1*]

[1] Center for Bionics, Korea Institute of Science and Technology (KIST), Seoul, 02792, Korea
[2] Korea University of Science and Technology, Daejeon, 34113, Korea
[3] Department of Electronics Engineering, Ewha Womans University, Seoul, 03760, Korea
[4] Department of Physiology, SBRI, Sungkyunkwan University School of Medicine, Suwon, 16419, Korea
[5] Department of Urology, Samsung Medical Center, Sungkyunkwan University School of Medicine, Seoul, 06351, Korea

† Equally credited first authors

*****Corresponding Author:
J-K. Francis Suh, Ph.D.
Center for Bionics, KIST
Hwarangno 14-gil 5, Sungbuk-gu
Seoul, 02792, Korea
Tel: +82-2-958-6651
Fax: +82-2-958-6446
E-Mail: jkfsuh@kist.re.kr

LEGEND FOR SUPPLEMENTARY VIDEO

Spontaneous vs. light-evoked voiding of transgenic animal model with ChR2-bladder
